# Cystic versus non-cystic prolactinoma: clinical, hormonal, radiological, and remission outcomes in Basrah

**DOI:** 10.3389/fendo.2025.1668255

**Published:** 2025-10-22

**Authors:** Khulood Abed Reman, Haider Ayad Alidrisi, Ali Hussain Ali Alhamza, Hassan Falah Alobaidy, Abbas Ali Mansour

**Affiliations:** Faiha Specialized Diabetes, Endocrine and Metabolism Center (FDEMC), University of Basrah, Basrah, Iraq

**Keywords:** pituitary adenoma, cystic prolactinoma, non-cystic prolactinoma, dopamine agonist, transsphenoidal surgery

## Abstract

**Introduction:**

Prolactinomas are the most common functional pituitary adenomas. Cystic prolactinomas, characterized by substantial cystic components on magnetic resonance imaging (MRI) scans, may exhibit different clinical behaviors and treatment outcomes. The aim of this study was to compare the clinical, hormonal, and radiological characteristics, and identify predictors of remission in patients with cystic versus non-cystic prolactinomas.

**Methods:**

This retrospective cohort study included 196 patients who were diagnosed with prolactinoma between January 2010 and January 2024. The patients were categorized into cystic (n=46) and non-cystic (n=150) groups based on their MRI characteristics. Data on clinical presentation, hormonal levels, imaging findings, treatment response, and remission predictors were analyzed.

**Results:**

Patients with cystic prolactinomas were younger and more likely to experience headaches. Lower follicular stimulating hormone levels were also observed in this group. Clinical symptom improvement over time did not differ significantly between the cystic and non-cystic prolactinomas at any follow-up interval. Prolactin levels declined similarly in both groups, and the percentage of patients who achieved a reduction in adenoma diameter did not differ significantly between the groups at any follow-up period. Remission rate was associated with lower baseline prolactin levels, early tumor shrinkage, and initial transsphenoidal surgery.

**Conclusion:**

Cystic morphology in prolactinomas does not compromise treatment response. Predictors of early remission can guide management strategies. Surgical intervention remains pivotal in selected cases.

## 1 Introduction

Prolactinomas are the most prevalent functional pituitary adenomas (PAs), accounting for approximately 40% of all PA cases (1). A prolactinoma is defined as cystic when more than 50% of its volume is filled with fluid (2). Historically, cystic prolactinomas were considered more resistant to medical therapy, often requiring surgical intervention (3). However, recent evidence suggests that they may respond favorably to dopamine agonists, similar to non-cystic prolactinomas (2).

Patients with prolactinomas may exhibit clinical symptoms of hormonal disturbances along the hypothalamic-pituitary axis, such as menstrual irregularities, galactorrhea, and infertility in women, and decreased libido and erectile dysfunction in men. They may also present with mass effect symptoms, including headache, dizziness, visual field impairments, and hypopituitarism (4).

Dopamine agonists (DAs) normalize prolactin levels and reduce tumor mass in most patients with prolactinomas, including those with large tumors and visual defects. DAs are typically the first-line therapy for prolactinomas owing to their effectiveness, ease of administration, and tolerability (1, 2, 4). Cystic changes in prolactinomas may reflect treatment effects rather than underlying pathology, and long-term cabergoline use has been associated with cystic degeneration of macroprolactinomas (5). In most patients, DAs effectively reduce the volume of cystic prolactinomas, with treatment responses comparable to those observed in non-cystic prolactinomas (6).

Patients with non-invasive, small adenomas who exhibit normal serum prolactin levels and significant tumor shrinkage after at least two years of low-dose cabergoline treatment (0.25–0.50 mg per week) have the highest likelihood of maintaining remission following withdrawal (7–9).

If no visible mass is detected on MRI, patients should be encouraged to discontinue treatment. Alternatively, dopamine agonists may be gradually tapered by reducing the dose and extending the dosing interval, aiming to identify the minimal effective dose needed to maintain normal serum prolactin levels (10).

When discontinuing dopamine agonist therapy, prolactin levels should be monitored every three months during the first year and annually thereafter. If hyperprolactinaemia recurs, a follow-up pituitary MRI may be warranted. In cases where treatment needs to be resumed due to recurrence, a second attempt at cabergoline withdrawal may be successful after an additional 2–3 years of therapy. This strategy may be especially beneficial for patients who maintain low prolactin levels while on treatment and have no detectable mass on pituitary MRI (11, 12).

The Endocrine Society Clinical Practice Guidelines, published in 2011, do not specifically address the management of cystic prolactinomas (13), whereas the Pituitary Society International Consensus Statement, published in 2023, indicates that cystic prolactinomas may respond to DA therapy. This approach may be a valid treatment option, particularly for patients who do not require urgent decompression of the optic chiasm (2).

Cystic pituitary adenomas may resemble Rathke’s cleft cysts, especially in the absence of a solid enhancing component found on magnetic resonance imaging (MRI) scans. However, features such as fluid-fluid levels, a hypointense T2-weighted rim, septations, an off-midline location, and the absence of an intracystic nodule (more common in Rathke’s cysts) may assist in their differentiation (14). Other cystic pituitary lesions include non-functional cystic adenomas, craniopharyngiomas, and arachnoid cysts (15).

Differentiating cystic from non-cystic prolactinomas is crucial because their different compositions influence treatment effectiveness and surgical approaches. Cystic prolactinomas may respond differently to dopamine agonist therapy, with some research showing lower remission rates and a higher need for surgery compared to solid tumors (16).

Surgical resection performed by highly skilled neurosurgeons offers a high cure rate for microadenomas and selected macroprolactinomas (Knosp 0–1), is cost-effective, and eliminates the need for long-term DA treatment. However, medical treatment remains the first-line therapy for tumors with low a possibility of surgical remission (Knosp grade ≥2). Surgery may be considered in patients with visual compromise, those with intolerance or resistance to long-term DA therapy, young women, or those planning pregnancy. Moreover, cerebrospinal fluid rhinorrhea may require surgical repair (2). The aim of this study was to compare the clinical, hormonal, and radiological characteristics, and identify predictors of remission in patients with cystic versus non-cystic prolactinomas.

## 2 Methods

### 2.1 Study design and population

This retrospective cohort study included patients with a confirmed diagnosis of prolactinoma based on clinical, biochemical, and radiological criteria, who were treated at Faiha Specialized Diabetes, Endocrine, and Metabolism Center (FDEMC) in Basrah, Southern Iraq, between January 2010 and January 2024. The study was approved by the FDEMC Ethical Committee (Approval No.: 56/35/22; Date: 19/03/2024), and all patients provided written informed consent to participating in this study.

### 2.2 Inclusion and exclusion criteria

This study included patients with confirmed hyperprolactinemia and pituitary adenoma on MRI scans. Based on imaging findings, patients were categorized into two groups: cystic and non-cystic adenomas. Cystic adenomas were defined as lesions with a cystic component occupying more than 50% of the tumor volume on an MRI scan (2). A total of 296 individuals were initially screened, of whom 100 patients were excluded due to incomplete or missing data, histopathological confirmation of Rathke’s cleft cyst, or cystic lesions not associated with prolactin secretion. Thus, 196 patients were included in the final analysis.

### 2.3 Data collection

Patient clinical records included information such as age at diagnosis; sex; marital status; main presenting symptoms; and prolactinoma-related symptoms, including galactorrhea, oligomenorrhea, hirsutism, acne, vasomotor symptoms, infertility, headache, visual field defects, gynecomastia, reduced libido, and erectile dysfunction. Presence of comorbid conditions, such as diabetes mellitus (DM) and hypertension was also recorded. Weight and height were measured, and body mass index (BMI) was calculated as weight in kilograms divided by height in meters squared (kg/m2). The records also included information on the source of referral and initial treatment approaches, which comprised medical therapy (either bromocriptine or cabergoline at a specified dosage), transsphenoidal surgery (TSS), or stereotactic radiosurgery.

Follow-up visit documentation was reviewed, including clinical improvement, laboratory parameters, radiological findings, and any changes to DA dose.

### 2.4 Biochemical analysis

Following an overnight fast of at least 8 hours, 10 mL of venous blood was collected in the morning (between 9:00 AM and 10:00 AM) to measure hormones including prolactin, follicle-stimulating hormone (FSH), luteinizing hormone (LH), total testosterone, estradiol (E2), adrenocorticotropic hormone, cortisol, dehydroepiandrosterone sulfate, thyroid-stimulating hormone, free thyroxine, and growth hormone. Prolactin levels were determined using an electrochemiluminescence immunoassay on the Roche Cobas e411 platform (Roche Holding, Basel, Switzerland).

### 2.5 Definition of variables and radiological assessment

The clinical terms used in this study, including galactorrhea, oligomenorrhea/amenorrhea, hirsutism, gynecomastia, reduced libido, erectile dysfunction, infertility, and visual field defect were defined in accordance with the standardized criteria established by the 2023 Pituitary Society Consensus (2).

Adenomas measuring 10 mm or more were classified as macroadenomas, while those smaller than 10 mm were termed microadenomas. Giant prolactinomas were defined as tumors 4 cm or larger (2).

For patients diagnosed with prolactinoma, pituitary MRI scans were obtained at diagnosis, and imaging studies were repeated at 3–6 months, 12 months, and subsequently at intervals beyond 24 months. These serial scans were used to monitor changes in adenoma diameter in response to medical treatment, to detect any signs or evidence of pituitary hemorrhage, optic chiasm compression, cavernous sinus invasion, or empty sella syndrome. All MRI scans in our study were performed using single standardized protocol. Furthermore, both baseline and follow up imagings were reviewed by the multidisciplinary team including endocrinologist and the same radiologist, which helped ensure consistency in imaging interpretation.

### 2.6 Follow-up and outcomes

Although all patients continued DA therapy during follow-up at FDEMC, the initial decision regarding treatment modality (medical or surgical) was made by the referring doctor prior to referral. Outcome measures for the evaluation of patients with prolactinoma on DA therapy were done at 6, 12, and 24 months, as well as at any time beyond 6 months after 24 months. The number reported at each follow-up visit represents the patients who showed up at that specific time point, as not all participants attended every visit due to the retrospective nature of the study. These measures include the following: (i) clinical improvement, defined as the resumption of regular cycles and/or achievement of pregnancy in married premenopausal women, and libido and erectile function in men; (ii) normalization of prolactin levels at any visit (< 425.5 mIU/L [20 ng/mL] for women, < 319.1 mIU/L [15 ng/mL] for men); (iii) radiological changes (adenoma diameter change, signs of pituitary hemorrhage, optic chiasm compression, cavernous sinus invasion, and empty sella syndrome) assessed via MRI scans at 12, 24, and beyond 24 months; and (iv) remission, defined as normalized prolactin levels and complete radiological disappearance of the tumor (2).

The number of patients included at each evaluation point represented those who showed up from the first visit to the center. Patients with persistently high prolactin levels or increasing prolactinoma diameter despite receiving the maximum dose of DA (cabergoline 3.5 mg per week) were referred for either TSS or gamma knife treatment. These non-medical options were only performed in those who accepted them. Patients who underwent TSS and/or gamma knife treatment, as well as those who discontinued DA therapy for any reason, were excluded from subsequent evaluations.

### 2.7 Statistical analysis

Clinical and demographic data were entered into Microsoft Excel and then analyzed using SPSS for Windows Version 23.0 (SPSS Inc., Chicago, USA). Continuous variables are presented as mean ± standard deviation (SD), while dichotomous variables are summarized as numbers and percentages.

Comparisons between the cystic and non-cystic groups were performed using the Student’s t-test for continuous variables and the chi-square or Fisher exact test for categorical variables. A P-value less than 0.05 was considered statistically significant.

## 3 Results

A total of 296 individuals were initially screened. Of these, 100 patients were excluded due to incomplete or missing data, histopathological confirmation of Rathke’s cleft cyst, or cystic lesions not associated with prolactin secretion. Therefore, 196 patients were included in the final analysis, including 46 (23.5%) with cystic prolactinomas and 150 (76.5%) with non-cystic prolactinomas. **Table 1** presents the baseline characteristics of both groups. Patients with cystic prolactinomas were significantly younger than those with non-cystic adenomas (mean age 28.0± 10.0 vs. 35.3  ± 12.3 years; P = 0.0003). The proportion of married individuals was also significantly lower in the cystic group (65.2% vs. 80.7%, P = 0.022).

**Table 1:** Initial clinical and demographic features of patients with cystic versus non-cystic prolactinoma.

Variable	Cystic prolactinoma	Non-cystic prolactinoma	*P* value
Age at diagnosis (years), mean ± SD	28.0 ± 10.0	35.3 ± 12.3	0.0003^1^
Age (years) median	24.0	34.0	
Gender (female)	31 (67.4)	95 (63.3)	0.6^2^
Married	30 (65.2)	121 (80.7)	0.02^2^
BMI (Kg/m^2^), mean ± SD	30.9 ± 8.8	32.8 ± 6.8	0.1^1^
DM	3 (6.5)	15 (10.1)	0.4^2^
Hypertension	9 (19.6)	30 (20.0)	0.9^2^
Galactorrhea	22 (47.8)	64 (42.7)	0.5^2^
Irregular menstruation (N 122)^4^	27 (87.1)	84 (92.3)	0.3^2^
Hirsutism (N 122)^4^	7 (22.6)	12 (13.2)	0.2^2^
Gynecomastia (N 70)^5^	2 (13.3)	8 (14.5)	0.9^2^
Reduced libido (N 70)^5^	11 (73.3)	48 (87.3)	0.1^2^
Erectile dysfunction (N 70)^5^	11 (73.3)	48 (87.3)	0.1^2^
Infertility (N 144)^6^	25 (83.3)	80 (70.2)	0.1^2^
Headache	38 (82.6)	100 (66.7)	0.03^2^
Visual field defect	12 (27.3)	33 (22.0)	0.4^2^
PRL (mIU/L), mean ± SD, (*N =* 185)	21359.5± 2963.3	28700± 84080.8	0.6^1^
PRL (mIU/L), median, (*N =* 185)	6872.3	6070.2	
PRL ≥ 10638.2 mIU/L (500 ng/mL), (*N =* 179)	8 (18.2)	38 (27.0)	0.2^2^
FSH (IU/L), mean ± SD, (*N =* 142)	3.2 ± 2.1	5.7 ± 9.3	0.01^1^
LH (IU/L), mean ± SD, (*N =* 141)	3.1 ± 3.5	4.4 ± 7.2	0.3^1^
TT (nmol/L), mean ± SD, (men) (*N = 42*)	4.3 ± 4.3	4.5 ± 4.1	0.9^1^
E2 (pmol/L), mean ± SD, (women) (*N =73)*	154.5± 146.8	201.1± 277.9	0.4^1^
ACTH (pmol/L), mean ± SD, (*N = 90*)	7.1± 4.4	6.9± 4.6	0.8^1^
Cortisol (nmol/L), mean ± SD, (*N =* 107)	405.5± 482.7	292.4± 146.2	0.1^1^
DHEA-s (µmol/L), mean ± SD, (*N = 100*)	8.9± 6.1	8.5± 6.6	0.8^1^
Adenoma (*N =* 183)^7^ Diameter (mm), mean ± SD	18.6 ± 12.4	15.8 ± 11.2	0.1^1^
Adenoma < 10 mm	11 (24.4)	46 (33.3)	
Adenoma 10–39 mm	29 (64.4)	85 (61.6)	0.2^2^
Adenoma ≥ 40 mm	5 (11.1)	7 (5.1)	
Cavernous invasion (Knosp2 and more)	13 (28.9)	44 (31.4)	0.7^2^
Optic chiasm abutment (*N =* 183)^7^	13 (28.9)	24 (17.4)	0.09^2^
Hydrocephalus (*N =* 183)^7^	1 (2.2)	1 (0.7)	0.4^3^
Empty Sella syndrome (*N =* 183)^7^	1 (2.2)	9 (6.5)	0.2^3^
Apoplexy (N 196)	3 (6.5)	4 (2.7)	0.2^3^
DA^8^	35 (76.1)	115 (76.7)	
TSS^9^	9 (19.6)	25 (16.7)	0.8^3^
Gamma knife	1 (2.2)	3 (2.0)	

^1^Independent student t test, ^2^Chi-Square test, ^3^Fisher Exact test, ^4^premenopausal women, ^5^men, ^6^married with childbearing potential, ^7^excluding 13 patients with initial TSS treatment and missed preoperative adenoma, ^8^later TSS was done for one cystic prolactinoma at 15 months and five non-cystic prolactinoma at 12, 15, and three at 48 months, and gamma knife for one non-cystic prolactinoma at 8 months, ^9^later gamma knife was for one cystic prolactinoma at 4 months and two non-cystic prolactinoma at 12 and 48 months.

SD, standard deviation; PRL, prolactin; FSH, follicular stimulating hormone; LH, luteinizing hormone; TT, total testosterone; E2, estradiol; ACTH, adrenocorticotrophic hormone; DHEA-s, dehydroepiandrosterone sulphate; DA, dopamine agonist; TSS, transsphenoidal pituitary surgery.

Reference ranges (SI Units): Prolactin: F 102–496 / M 86–324 mIU/L; FSH: Foll 3.5–12.5 / Luteal 1.5–7.0 / M 1.5–12.4 IU/L; LH: Foll 2.4–12.6 / Luteal 1.0–11.4 / M 1.7–8.6 IU/L; Total Testosterone: M 8.6–29.0 / F 0.3–2.4 nmol/L; E2: Foll 68–530 / Luteal 91–730 / M <160 pmol/L; ACTH: 1.6–13.9 pmol/L; Cortisol: 140–690 nmol/L; DHEAS: M 4.1–12.1 / F 2.7–9.2 µmol/L.

Headache was reported more frequently among patients with cystic prolactinomas (82.6%) than among those without (66.7%, P = 0.032). Additionally, the cystic group exhibited significantly lower baseline FSH levels (3.2± 2.1 vs. 5.7 ± 9.3IU/L, P = 0.011). No statistically significant differences were observed between the two groups in terms of sex distribution, BMI, presence of comorbidities (DM or hypertension), baseline prolactin levels, other hormonal parameters (LH, E2, and testosterone), adenoma size, cavernous sinus invasion, optic chiasm compression, or initial treatment modalities.

**Figure 1:**
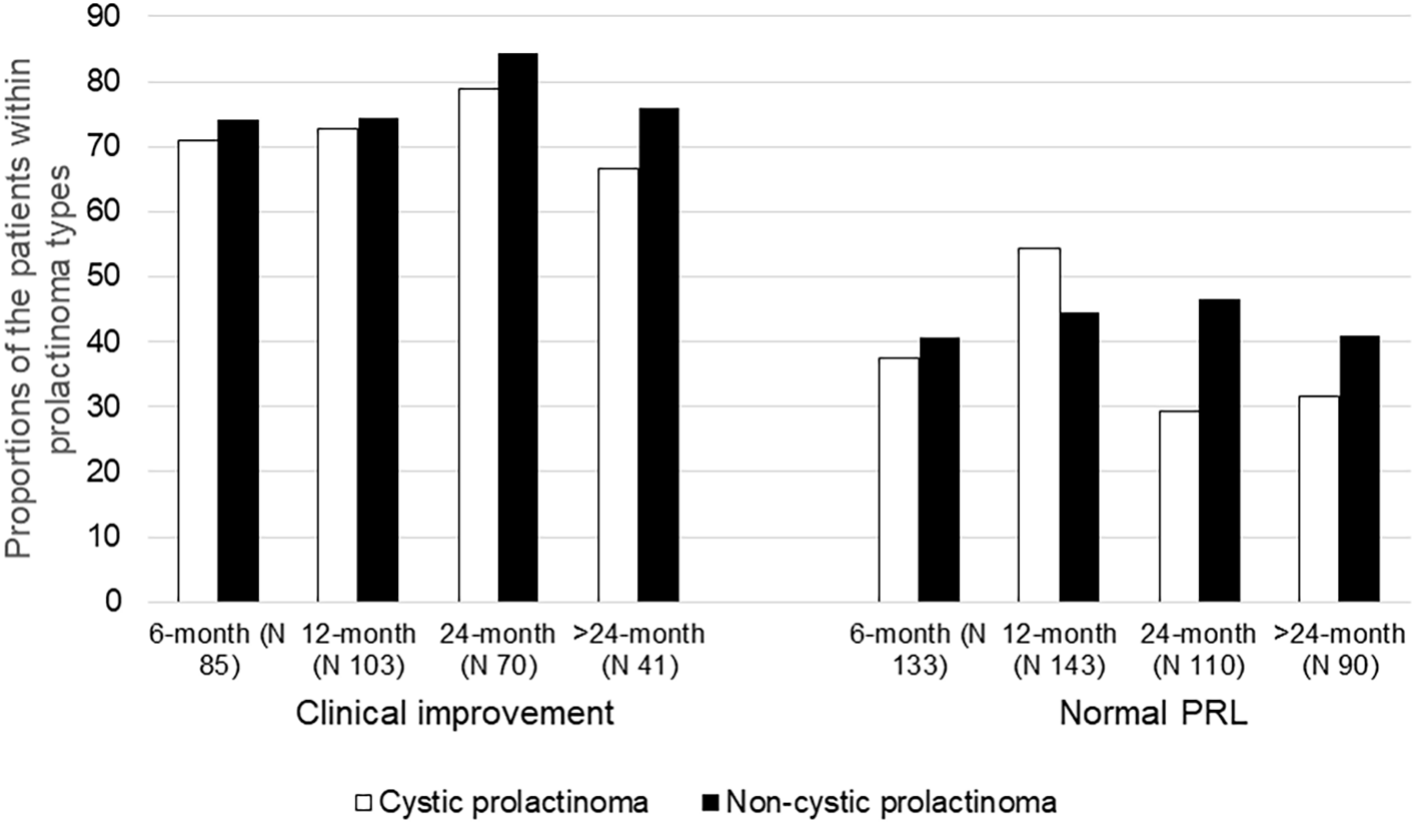
Comparison of clinical improvement and normalization of prolactin levels between cystic and non-cystic prolactinoma patients over the follow-up period. All chi-square P values for comparisons exceeded 0.05. The number reported at each follow-up visit represents the patients who showed up at that specific time point.

As shown in **Figure 1**, no statistically significant difference was observed in the rate of clinical symptom improvement between patients with cystic and non-cystic prolactinomas across all follow-up intervals, and at all follow up points, prolactin level decline was similar between the two groups (P>0.05).

**Figure 2:**
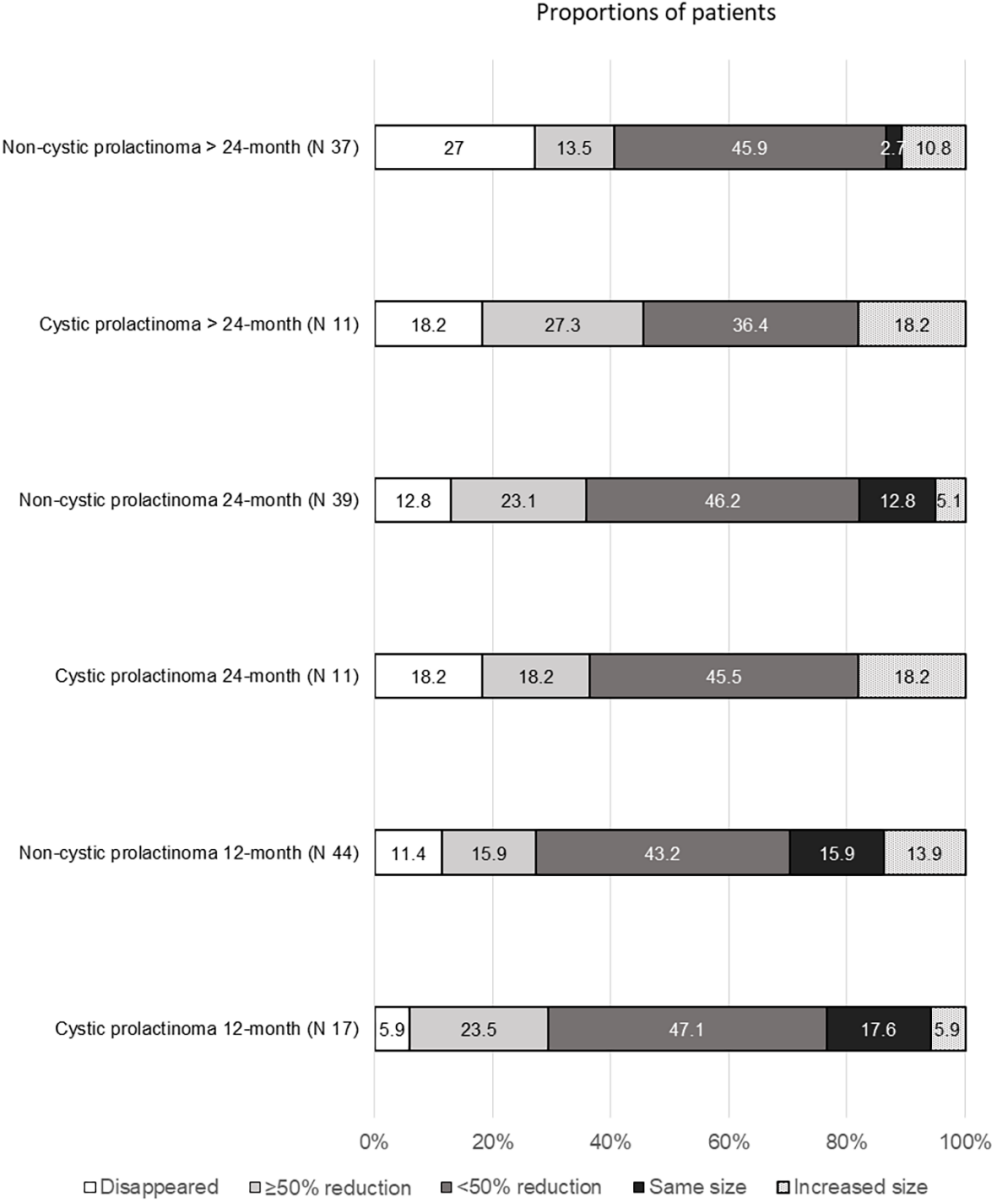
Comparison of radiological changes between cystic and non-cystic prolactinoma groups. All chi-square P values for comparisons exceeded 0.05 at 12, 24, and beyond 24 months (excluding patients with initial TSS). The number reported at each follow-up visit represents the patients who showed up at that specific time point.

Radiological evaluation of adenoma shrinkage at 12, 24, and beyond 24 months (excluding that for patients treated with primary surgery) is shown in **Figure 2**. The proportion of patients who achieved adenoma diameter reduction did not differ significantly between the groups at any follow-up point.

**Table 2** details the results of the univariate analysis for factors associated with remission at 2 years. Patients with baseline prolactin levels below 10638.2 mIU/L (500 ng/mL) had a significantly higher likelihood of achieving normalized prolactin levels and adenoma disappearance than those with prolactin levels exceeding 10638.2 mIU/L (500 ng/mL) (33.8% vs. 12.9%, P = 0.03).

**Table 2:** Univariate analysis of factors associated with remission of prolactinoma after 2 years of follow-up.

Variable	Normal PRL and disappeared adenoma N (%)		
	Yes	No	*P* value^1^
Men	14 (35.9)	25 (64.1)	0.1
Women	12 (21.1)	45 (78.9)	
Age <45 years	21 (28.0)	54 (72.0)	0.7
Age ≥45 years	5 (23.8)	16 (76.2)	
BMI <30 kg/m2	6 (18.2)	27 (81.8)	0.1
BMI ≥30 kg/m2	20 (32.8)	41 (67.2)	
PRL <10638.2 mIU/L (500 ng/mL)	22 (33.8)	43 (66.2)	0.03
PRL ≥10638.2 mIU/L (500 ng/mL)	4 (12.9)	27 (87.1)	
Adenoma <10 mm	6 (30.0)	14 (70.0)	0.7
Adenoma ≥ 10 mm	20 (26.7)	55 (73.3)	
Adenoma <40 mm	24 (27.9)	62 (72.1)	0.9^2^
Adenoma ≥ 40 mm	2 (22.2)	7 (77.8)	
Cystic prolactinoma	5 (19.2)	21 (80.8)	0.2
Non-cystic prolactinoma	21 (30.0)	49 (70.0)	
Initial TSS	9 (52.9)	8 (47.1)	0.008
No initial TSS	17 (21.5)	62 (78.5)	
12-month normal PRL	16 (35.6)	29 (64.4)	0.03
12-month high PRL	7 (15.9)	37 (84.1)	
12-month ≥50% adenoma shrinkage	10 (50.0)	10 (50.0)	0.006
12-month <50% adenoma shrinkage	7 (16.7)	35 (83.3)	

^1^chi-square P value, ^2^Fisher exact P value.

PRL, prolactin; TSS, transsphenoidal pituitary surgery.

TSS was associated with improved outcomes. More than half of the patients initially managed with surgery achieved the target outcome (52.9% vs. 21.5%, P = 0.008).

Patients with normalized prolactin levels at 12 months were significantly more likely to achieve remission (35.6 vs. 15.9%, P = 0.03) compared with their counterparts. Similarly, patients who experienced 50% adenoma shrinkage at 12 months had a markedly higher rate of complete adenoma resolution after 2 years compared with those with less than 50% adenoma shrinkage (50.0% vs. 16.7%, P = 0.006).

Factors such as sex, age, BMI, initial adenoma size, and presence of cystic components were not significantly associated with remission achievement.

A multinominal regression analysis was performed to detect the independent factors associated with prolactinoma remission as shown in **Table 3**. The achievement of normal PRL and or adenoma shrinkage equal or more than 50% were significantly associated with remission and independently of the initial PRL level and the initial treatment modality (TSS versus DA).

**Table 3:** Multinominal regression analysis for the effects of baseline PRL, initial TSS, normalization of PRL at 12-month, and adenoma shrinkage equal of more than 50% at 12-month on the remission of prolactinoma.

Predictors	OR (95% CI)	*P* value
PRL <10638.2 mIU/L (500 ng/mL)	2.7 (0.6-11.4)	0.1
Initial TSS	0.5 (0.1-2.5)	0.4
12-month normal PRL	4.7 (1.0-20.7)	0.03
12-month ≥50% adenoma shrinkage	4.7 (1.2-18.1)	0.02

PRL, prolactin; TSS, transsphenoidal pituitary surgery; OR, odd ratio; CI, confidence interval.

## 4 Discussion

To our knowledge, this is the first such study conducted in Iraq. The only related research from the same center in Basrah, Southern Iraq, was focused on all types of pituitary lesions, and revealed that prolactinoma constitutes 26.9% of pituitary adenomas. This finding was explained by referral bias because patients with acromegaly are typically referred to a tertiary center, while prolactinoma and non-functioning pituitary adenomas are often managed by gynecologists and neurosurgeons (17). In addition, a multicenter retrospective study in Al-Ain, United Arab Emirates, showed prolactinomas to be the most common sellar masses, constituting 56.7% of all pituitary adenomas and 51.1% of all sellar lesions (18).

Our data showed that more patients with cystic prolactinomas were diagnosed at a younger age than were those with non-cystic adenomas. This may reflect an earlier disease onset or more acute symptoms associated with cystic changes. This finding is consistent with the study by Su et al. which also reported that cystic prolactinomas patients were significantly younger at diagnosis (19). The Pituitary Society Consensus notes that prolactinomas in younger individuals often have different characteristics than those in older patients, although it does not specifically mention cystic prolactinomas (2).

In our study, the proportion of married individuals was significantly lower in the cystic group. While no previous studies were found to directly address this specific observation, this finding may be indirectly related to the younger age of these patients. As observed in our study, cystic prolactinomas tended to occur in younger individuals who may not have reached typical marital age. Additionally, hyperprolactinemia in younger patients may lead to symptoms such as menstrual irregularities, infertility, or decreased libido, potentially impacting relationship and delaying marriage.

Headache occurred more frequently among patients with cystic prolactinoma than among those with non-cystic prolactinomas, this may be attributed to the mass effect of the cystic component, which can cause increased pressure on surrounding structures. Joa et al., revealed that patients with prolactinomas had the highest headache incidence (83%), although no statistical difference in headache occurrence was observed between cystic and non-cystic adenomas (20). However, the study showed that biochemical-neuroendocrine factors (hormone secretion) may play a role in headache pathogenesis, independent of structural effects such as cyst formation.

Baseline FSH levels were significantly lower in the cystic group in the present study. Low FSH levels in cystic prolactinoma patients may be attributed to their younger age, as in our study, cystic prolactinomas occurred in younger individuals, whereas solid prolactinomas were seen in peri-and postmenopausal patients. Additionally, the cystic component itself may exert a mass effect on the hypothalamic-pituitary axis, contributing further to FSH suppression. This observation aligns with the findings of Su et al. (2023). study, which is a retrospective study of 141 patients with prolactinoma (41 cystic and 79 solid macroprolactinomas) revealed that cystic prolactinomas often present with larger tumor sizes and higher preoperative prolactin levels, which are independent predictors of hypogonadism (19).

Baseline prolactin level and adenoma size showed no significant differences across groups, this may be related to individual tumor characteristics, including the presence of hormonally active solid components within some cystic tumor. However. It contrasts with the belief that cystic prolactinomas secrete less prolactin owing to reduced cellular content and that solid macroprolactinomas are larger than cystic or solid microprolactinomas (19). The presence of cystic components may affect prolactin secretion, resulting in lower baseline levels in some cases. Therefore, baseline prolactin levels can vary between patients with cystic and non-cystic prolactinomas.

No significant differences in clinical symptom improvement, prolactin normalization, or adenoma diameter reduction were observed between the cystic and non-cystic groups at any follow-up point. This suggests that the presence of cystic changes does not negatively affect response to medical or surgical treatments. This finding aligns with that of a study showing that DAs are effective in reducing the volume of cystic prolactinomas in most patients, and treatment responses were similar to those frequently observed in patients with non-cystic prolactinomas (6). This finding is consistent with the literature, revealing that DA therapy may be an effective and safe treatment option for a considerable proportion of patients with cystic prolactinomas (21).

In our study, baseline prolactin levels of < 10638.2 mIU/L (500 ng/mL) were associated with remission at 2 years in the univariate analysis. However, this association was not retained in the multivariate analysis, suggesting that while lower initial prolactin levels may indicate a more favorable prognosis, they are not independently predictive of remission when adjusted for other factors. A retrospective study conducted to evaluate 142 patients with prolactinomas treated exclusively with DAs indicated that patients with lower initial serum prolactin levels, particularly those with microadenomas, had a higher likelihood of successful therapy withdrawal and remission. Macroprolactinomas are more prone to relapse than are microprolactinomas. While the recurrence group had higher median initial serum prolactin levels, this difference did not reach statistical significance (22).

While specific studies directly linking adenoma shrinkage of more than 50% at 12 months to remission at 2 years are limited, a study focused on macroprolactinomas revealed that patients who experienced significant adenoma shrinkage within the first 3 months of cabergoline treatment were more likely to have a favorable long-term response, indicating that early adenoma size reduction could predict sustained remission (23).

Patients who underwent initial TSS had a significantly higher remission rate than those managed with medical treatment in the univariate analysis. However, this association did not remain significant in the multivariate analysis. This may suggest that the observed remission was not necessarily a direct result of surgery itself, but rather due to other contributing factors such as tumor regression in size. A long-term follow-up study showed that first-line surgery achieved remission in 72% and 45% of patients with microprolactinomas and macroprolactinomas, respectively. This study highlighted that patients with Knosp grade 0 adenomas had better outcomes, suggesting that the degree of cavernous sinuses invasion plays a role in surgical success (24). A retrospective study involving 41 patients with cystic prolactinomas showed early postoperative remission in 65.83% of cases and a long-term remission rate of 58.54% over an average follow-up period of approximately 44 months. This study identified adenoma size and preoperative prolactin level as significant predictors of remission (13).

This study has some limitations. First, it was a single-center study, reflecting its clinical patterns and management practices. While these findings contribute to a better understanding of prolactinoma subtypes, further multicenter studies are required to validate these findings. Second, this study was retrospective.

In conclusion, the presence of cystic components in prolactinomas does not negatively affect treatment outcomes. No significant difference was observed in clinical symptom improvement between cystic and non-cystic prolactinomas at any follow-up interval. Both groups showed comparable declines in prolactin levels, and the proportion of patients with reduced adenoma size was similar across all follow-up periods. Identifying early predictors of remission can help optimize patient management. Surgical intervention plays a crucial role in achieving remission in carefully selected patients.

## Data Availability

The original contributions presented in the study are included in the article/supplementary material. Further inquiries can be directed to the corresponding author.
